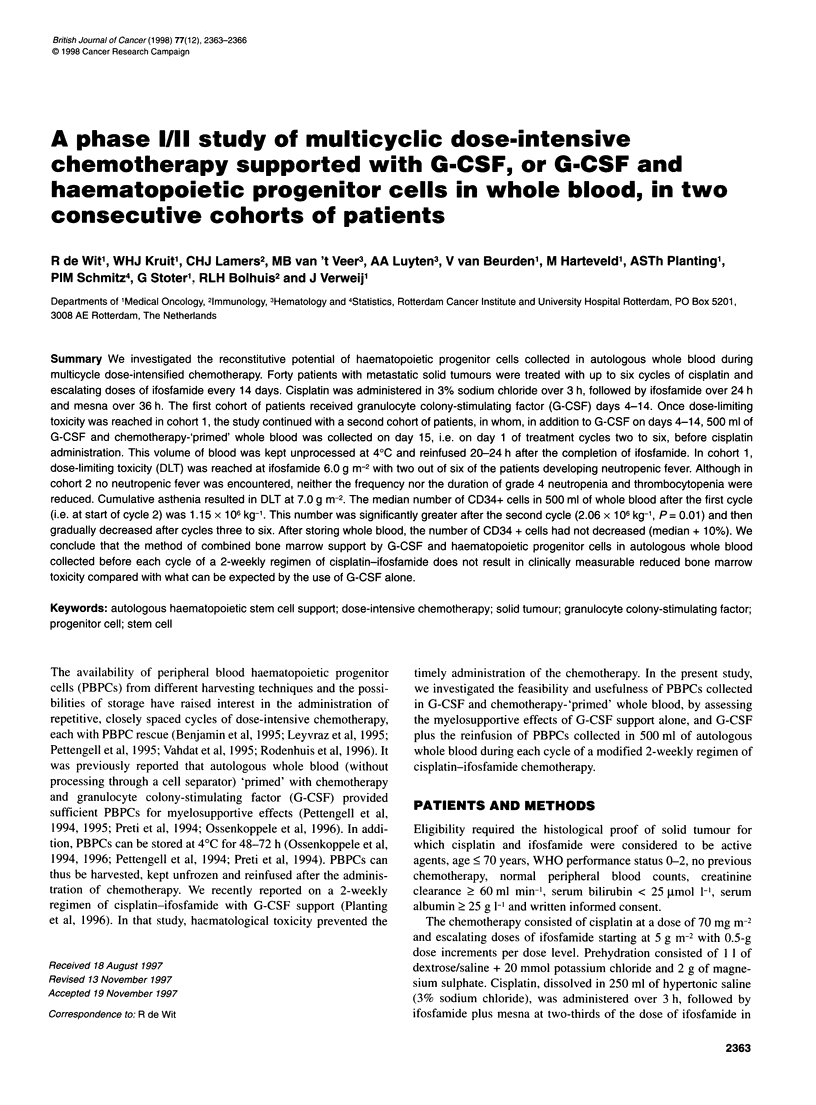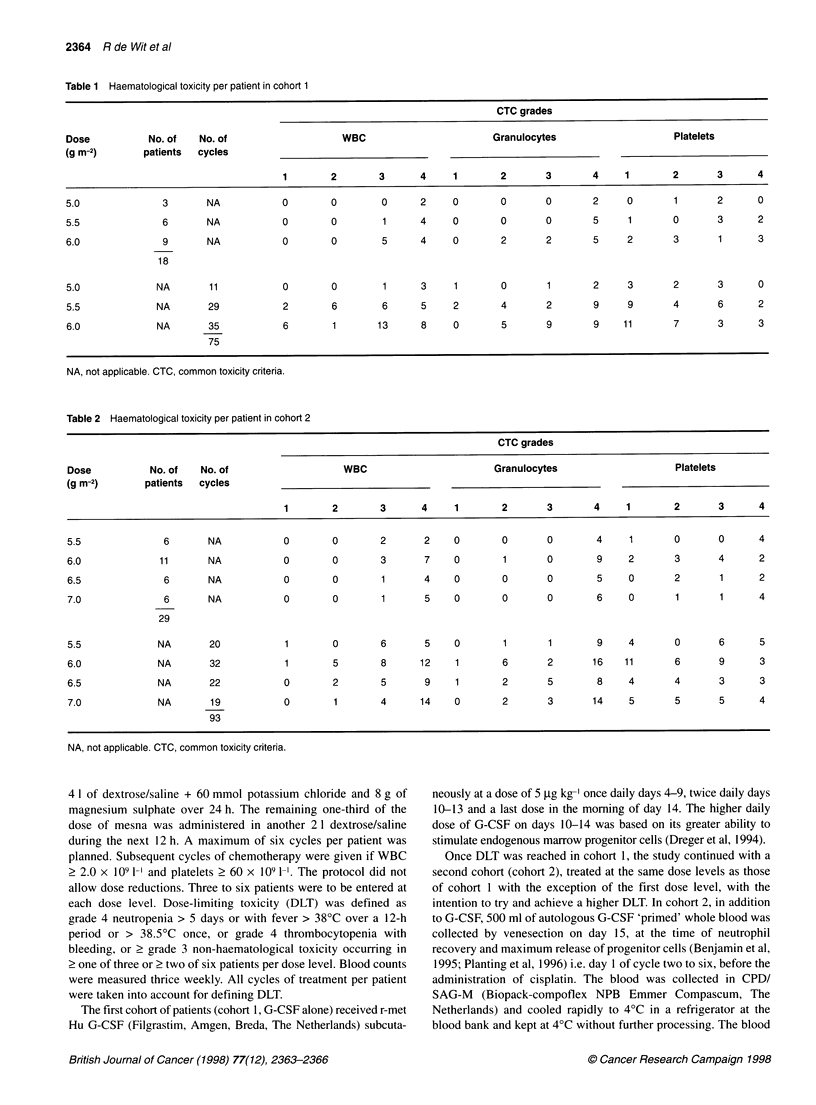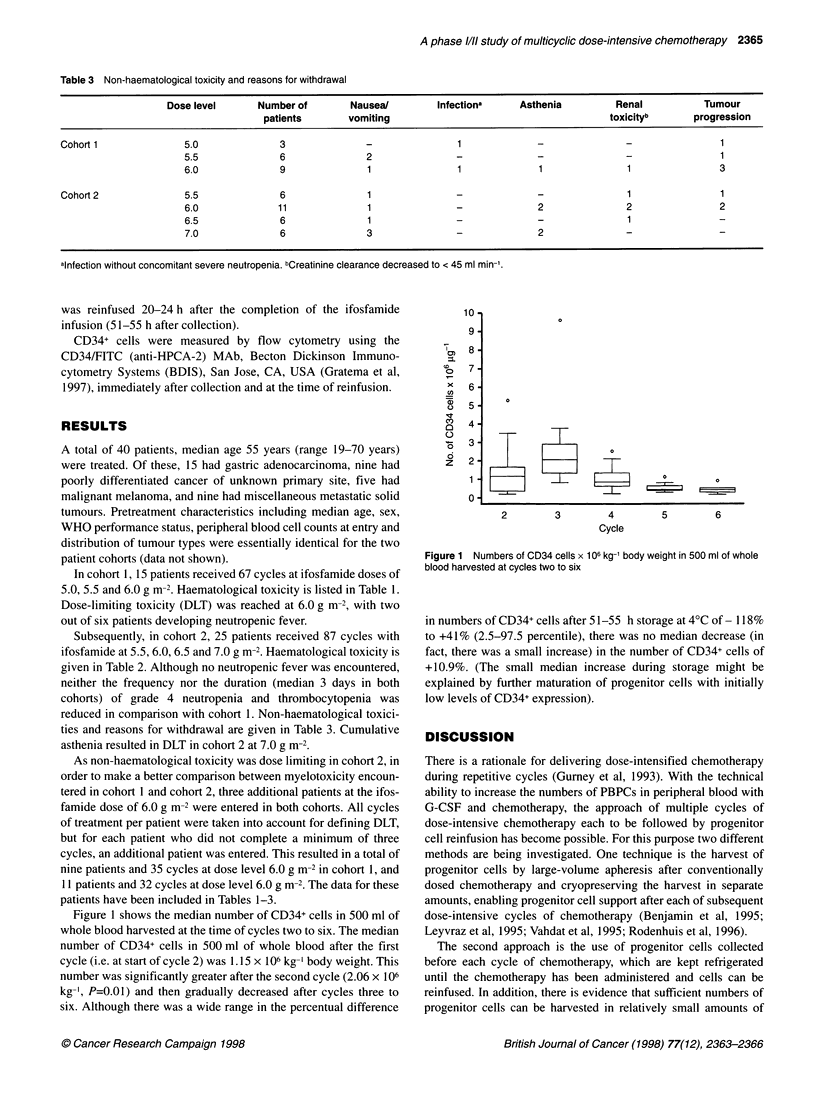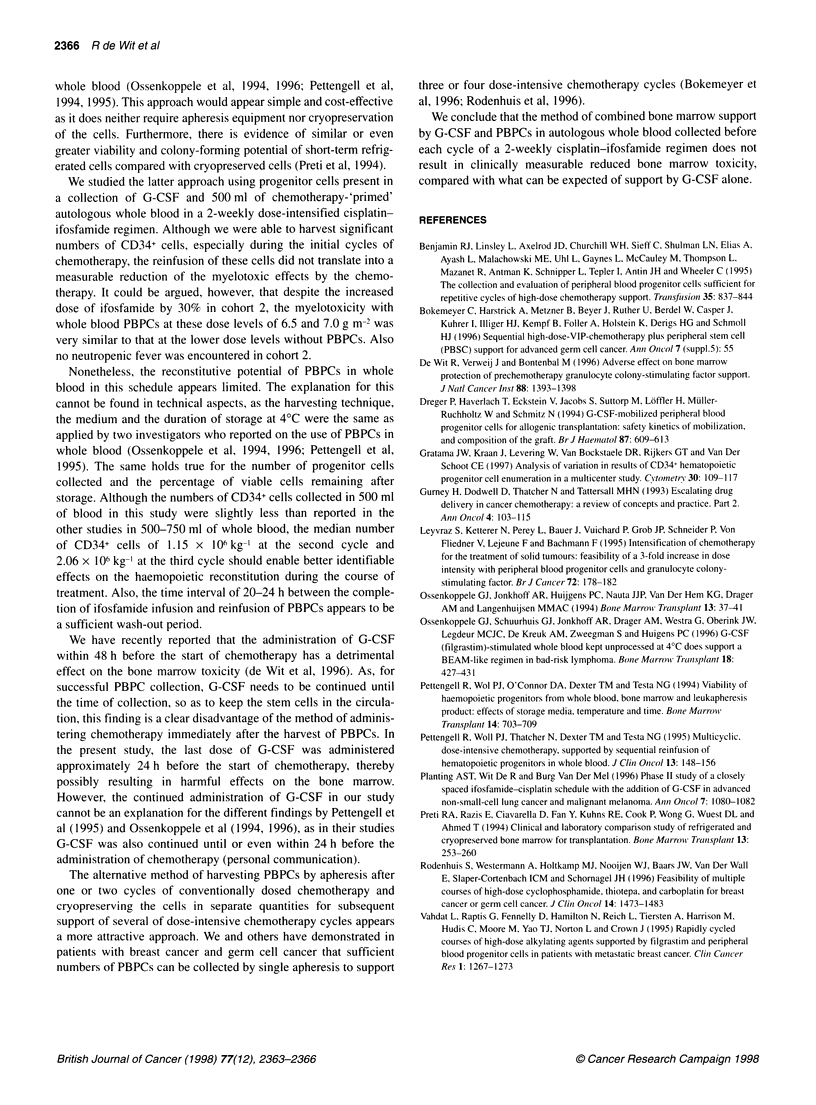# A phase I/II study of multicyclic dose-intensive chemotherapy supported with G-CSF, or G-CSF and haematopoietic progenitor cells in whole blood, in two consecutive cohorts of patients.

**DOI:** 10.1038/bjc.1998.392

**Published:** 1998-06

**Authors:** R. de Wit, W. H. Kruit, C. H. Lamers, M. B. van 't Veer, A. A. Luyten, V. van Beurden, M. Harteveld, A. S. Planting, P. I. Schmitz, G. Stoter, R. L. Bolhuis, J. Verweij

**Affiliations:** Department of Medical Oncology, Rotterdam Cancer Institute and University Hospital Rotterdam, The Netherlands.

## Abstract

We investigated the reconstitutive potential of haematopoietic progenitor cells collected in autologous whole blood during multicycle dose-intensified chemotherapy. Forty patients with metastatic solid tumours were treated with up to six cycles of cisplatin and escalating doses of ifosfamide every 14 days. Cisplatin was administered in 3% sodium chloride over 3 h, followed by ifosfamide over 24 h and mesna over 36 h. The first cohort of patients received granulocyte colony-stimulating factor (G-CSF) days 4-14. Once dose-limiting toxicity was reached in cohort 1, the study continued with a second cohort of patients, in whom, in addition to G-CSF on days 4-14, 500 ml of G-CSF and chemotherapy-'primed' whole blood was collected on day 15, i.e. on day 1 of treatment cycles two to six, before cisplatin administration. This volume of blood was kept unprocessed at 4 degrees C and reinfused 20-24 h after the completion of ifosfamide. In cohort 1, dose-limiting toxicity (DLT) was reached at ifosfamide 6.0 g m(-2) with two out of six of the patients developing neutropenic fever. Although in cohort 2 no neutropenic fever was encountered, neither the frequency nor the duration of grade 4 neutropenia and thrombocytopenia were reduced. Cumulative asthenia resulted in DLT at 7.0 g m(-2). The median number of CD34+ cells in 500 ml of whole blood after the first cycle (i.e. at start of cycle 2) was 1.15 x 10(6) kg(-1). This number was significantly greater after the second cycle (2.06 x 10(6) kg(-1), P = 0.01) and then gradually decreased after cycles three to six. After storing whole blood, the number of CD34+ cells had not decreased (median + 10%). We conclude that the method of combined bone marrow support by G-CSF and haematopoietic progenitor cells in autologous whole blood collected before each cycle of a 2-weekly regimen of cisplatin-ifosfamide does not result in clinically measurable reduced bone marrow toxicity compared with what can be expected by the use of G-CSF alone.


British Journal of Cancer (1998) 77(12), 2363–2366
© 1998 Cancer Research Campaign

### A phase I/II study of multicyclic dose-intensive chemotherapy supported with G-CSF, or G-CSF and haematopoietic progenitor cells in whole blood, in two consecutive cohorts of patients

**R de Wit[1], WHJ Kruit[1], CHJ Lamers[2], MB van 't Veer[3], AA Luyten[3], V van Beurden[1], M Harteveld[1], ASTh Planting[1], PIM Schmitz[4], G Stoter[1], RLH Bolhuis[2] and J Verweij[1]**

Departments of [1]Medical Oncology, [2]Immunology, [3]Hematology and [4]Statistics, Rotterdam Cancer Institute and University Hospital Rotterdam, PO Box 5201, 3008 AE Rotterdam, The Netherlands

**Summary** We investigated the reconstitutive potential of haematopoietic progenitor cells collected in autologous whole blood during multicycle dose-intensified chemotherapy. Forty patients with metastatic solid tumours were treated with up to six cycles of cisplatin and escalating doses of ifosfamide every 14 days. Cisplatin was administered in 3% sodium chloride over 3 h, followed by ifosfamide over 24 h and mesna over 36 h. The first cohort of patients received granulocyte colony-stimulating factor (G-CSF) days 4–14. Once dose-limiting toxicity was reached in cohort 1, the study continued with a second cohort of patients, in whom, in addition to G-CSF on days 4–14, 500 ml of G-CSF and chemotherapy-'primed' whole blood was collected on day 15, i.e. on day 1 of treatment cycles two to six, before cisplatin administration. This volume of blood was kept unprocessed at 4°C and reinfused 20–24 h after the completion of ifosfamide. In cohort 1, dose-limiting toxicity (DLT) was reached at ifosfamide 6.0 g $m^{-2}$ with two out of six of the patients developing neutropenic fever. Although in cohort 2 no neutropenic fever was encountered, neither the frequency nor the duration of grade 4 neutropenia and thrombocytopenia were reduced. Cumulative asthenia resulted in DLT at 7.0 g $m^{-2}$. The median number of CD34+ cells in 500 ml of whole blood after the first cycle (i.e. at start of cycle 2) was $1.15 \times 10^6$ $kg^{-1}$. This number was significantly greater after the second cycle ($2.06 \times 10^6$ $kg^{-1}$, $P = 0.01$) and then gradually decreased after cycles three to six. After storing whole blood, the number of CD34 + cells had not decreased (median + 10%). We conclude that the method of combined bone marrow support by G-CSF and haematopoietic progenitor cells in autologous whole blood collected before each cycle of a 2-weekly regimen of cisplatin–ifosfamide does not result in clinically measurable reduced bone marrow toxicity compared with what can be expected by the use of G-CSF alone.

**Keywords:** autologous haematopoietic stem cell support; dose-intensive chemotherapy; solid tumour; granulocyte colony-stimulating factor; progenitor cell; stem cell

The availability of peripheral blood haematopoietic progenitor cells (PBPCs) from different harvesting techniques and the possibilities of storage have raised interest in the administration of repetitive, closely spaced cycles of dose-intensive chemotherapy, each with PBPC rescue (Benjamin et al, 1995; Leyvraz et al, 1995; Pettengell et al, 1995; Vahdat et al, 1995; Rodenhuis et al, 1996). It was previously reported that autologous whole blood (without processing through a cell separator) 'primed' with chemotherapy and granulocyte colony-stimulating factor (G-CSF) provided sufficient PBPCs for myelosupportive effects (Pettengell et al, 1994, 1995; Preti et al, 1994; Ossenkoppele et al, 1996). In addition, PBPCs can be stored at 4°C for 48–72 h (Ossenkoppele et al, 1994, 1996; Pettengell et al, 1994; Preti et al, 1994). PBPCs can thus be harvested, kept unfrozen and reinfused after the administration of chemotherapy. We recently reported on a 2-weekly regimen of cisplatin–ifosfamide with G-CSF support (Planting et al, 1996). In that study, haematological toxicity prevented the timely administration of the chemotherapy. In the present study, we investigated the feasibility and usefulness of PBPCs collected in G-CSF and chemotherapy-'primed' whole blood, by assessing the myelosupportive effects of G-CSF support alone, and G-CSF plus the reinfusion of PBPCs collected in 500 ml of autologous whole blood during each cycle of a modified 2-weekly regimen of cisplatin–ifosfamide chemotherapy.

*Received 18 August 1997*
*Revised 13 November 1997*
*Accepted 19 November 1997*

*Correspondence to:* R de Wit

### PATIENTS AND METHODS

Eligibility required the histological proof of solid tumour for which cisplatin and ifosfamide were considered to be active agents, age ≤ 70 years, WHO performance status 0–2, no previous chemotherapy, normal peripheral blood counts, creatinine clearance ≥ 60 ml $min^{-1}$, serum bilirubin < 25 μmol $l^{-1}$, serum albumin ≥ 25 g $l^{-1}$ and written informed consent.

The chemotherapy consisted of cisplatin at a dose of 70 mg $m^{-2}$ and escalating doses of ifosfamide starting at 5 g $m^{-2}$ with 0.5-g dose increments per dose level. Prehydration consisted of 1 l of dextrose/saline + 20 mmol potassium chloride and 2 g of magnesium sulphate. Cisplatin, dissolved in 250 ml of hypertonic saline (3% sodium chloride), was administered over 3 h, followed by ifosfamide plus mesna at two-thirds of the dose of ifosfamide in

**Table 1** Haematological toxicity per patient in cohort 1

| Dose (g $m^{-2}$) | No. of patients | No. of cycles | CTC grades | | | | | | | | | | | |
|---|---|---|---|---|---|---|---|---|---|---|---|---|---|---|
| | | | WBC | | | | Granulocytes | | | | Platelets | | | |
| | | | 1 | 2 | 3 | 4 | 1 | 2 | 3 | 4 | 1 | 2 | 3 | 4 |
| 5.0 | 3 | NA | 0 | 0 | 0 | 2 | 0 | 0 | 0 | 2 | 0 | 1 | 2 | 0 |
| 5.5 | 6 | NA | 0 | 0 | 1 | 4 | 0 | 0 | 0 | 5 | 1 | 0 | 3 | 2 |
| 6.0 | 9 | NA | 0 | 0 | 5 | 4 | 0 | 2 | 2 | 5 | 2 | 3 | 1 | 3 |
| | 18 | | | | | | | | | | | | | |
| 5.0 | NA | 11 | 0 | 0 | 1 | 3 | 1 | 0 | 1 | 2 | 3 | 2 | 3 | 0 |
| 5.5 | NA | 29 | 2 | 6 | 6 | 5 | 2 | 4 | 2 | 9 | 9 | 4 | 6 | 2 |
| 6.0 | NA | 35 | 6 | 1 | 13 | 8 | 0 | 5 | 9 | 9 | 11 | 7 | 3 | 3 |
| | | 75 | | | | | | | | | | | | |

NA, not applicable. CTC, common toxicity criteria.

**Table 2** Haematological toxicity per patient in cohort 2

| Dose (g $m^{-2}$) | No. of patients | No. of cycles | CTC grades | | | | | | | | | | | |
|---|---|---|---|---|---|---|---|---|---|---|---|---|---|---|
| | | | WBC | | | | Granulocytes | | | | Platelets | | | |
| | | | 1 | 2 | 3 | 4 | 1 | 2 | 3 | 4 | 1 | 2 | 3 | 4 |
| 5.5 | 6 | NA | 0 | 0 | 2 | 2 | 0 | 0 | 0 | 4 | 1 | 0 | 0 | 4 |
| 6.0 | 11 | NA | 0 | 0 | 3 | 7 | 0 | 1 | 0 | 9 | 2 | 3 | 4 | 2 |
| 6.5 | 6 | NA | 0 | 0 | 1 | 4 | 0 | 0 | 0 | 5 | 0 | 2 | 1 | 2 |
| 7.0 | 6 | NA | 0 | 0 | 1 | 5 | 0 | 0 | 0 | 6 | 0 | 1 | 1 | 4 |
| | 29 | | | | | | | | | | | | | |
| 5.5 | NA | 20 | 1 | 0 | 6 | 5 | 0 | 1 | 1 | 9 | 4 | 0 | 6 | 5 |
| 6.0 | NA | 32 | 1 | 5 | 8 | 12 | 1 | 6 | 2 | 16 | 11 | 6 | 9 | 3 |
| 6.5 | NA | 22 | 0 | 2 | 5 | 9 | 1 | 2 | 5 | 8 | 4 | 4 | 3 | 3 |
| 7.0 | NA | 19 | 0 | 1 | 4 | 14 | 0 | 2 | 3 | 14 | 5 | 5 | 5 | 4 |
| | | 93 | | | | | | | | | | | | |

NA, not applicable. CTC, common toxicity criteria.

4 l of dextrose/saline + 60 mmol potassium chloride and 8 g of magnesium sulphate over 24 h. The remaining one-third of the dose of mesna was administered in another 2 l dextrose/saline during the next 12 h. A maximum of six cycles per patient was planned. Subsequent cycles of chemotherapy were given if WBC $\geq 2.0 \times 10^9\ l^{-1}$ and platelets $\geq 60 \times 10^9\ l^{-1}$. The protocol did not allow dose reductions. Three to six patients were to be entered at each dose level. Dose-limiting toxicity (DLT) was defined as grade 4 neutropenia > 5 days or with fever > 38°C over a 12-h period or > 38.5°C once, or grade 4 thrombocytopenia with bleeding, or ≥ grade 3 non-haematological toxicity occurring in ≥ one of three or ≥ two of six patients per dose level. Blood counts were measured thrice weekly. All cycles of treatment per patient were taken into account for defining DLT.

The first cohort of patients (cohort 1, G-CSF alone) received r-met Hu G-CSF (Filgrastim, Amgen, Breda, The Netherlands) subcutaneously at a dose of 5 $\mu g\ kg^{-1}$ once daily days 4–9, twice daily days 10–13 and a last dose in the morning of day 14. The higher daily dose of G-CSF on days 10–14 was based on its greater ability to stimulate endogenous marrow progenitor cells (Dreger et al, 1994).

Once DLT was reached in cohort 1, the study continued with a second cohort (cohort 2), treated at the same dose levels as those of cohort 1 with the exception of the first dose level, with the intention to try and achieve a higher DLT. In cohort 2, in addition to G-CSF, 500 ml of autologous G-CSF 'primed' whole blood was collected by venesection on day 15, at the time of neutrophil recovery and maximum release of progenitor cells (Benjamin et al, 1995; Planting et al, 1996) i.e. day 1 of cycle two to six, before the administration of cisplatin. The blood was collected in CPD/SAG-M (Biopack-compoflex NPB Emmer Compascum, The Netherlands) and cooled rapidly to 4°C in a refrigerator at the blood bank and kept at 4°C without further processing. The blood

*© Cancer Research Campaign 1998*

**Table 3** Non-haematological toxicity and reasons for withdrawal

| | Dose level | Number of patients | Nausea/ vomiting | Infection[a] | Asthenia | Renal toxicity[b] | Tumour progression |
|---|---|---|---|---|---|---|---|
| Cohort 1 | 5.0 | 3 | – | 1 | – | – | 1 |
| | 5.5 | 6 | 2 | – | – | – | 1 |
| | 6.0 | 9 | 1 | 1 | 1 | 1 | 3 |
| Cohort 2 | 5.5 | 6 | 1 | – | – | 1 | 1 |
| | 6.0 | 11 | 1 | – | 2 | 2 | 2 |
| | 6.5 | 6 | 1 | – | – | 1 | – |
| | 7.0 | 6 | 3 | – | 2 | – | – |

[a]Infection without concomitant severe neutropenia. [b]Creatinine clearance decreased to < 45 ml min$^{-1}$.

was reinfused 20–24 h after the completion of the ifosfamide infusion (51–55 h after collection).

CD34$^+$ cells were measured by flow cytometry using the CD34/FITC (anti-HPCA-2) MAb, Becton Dickinson Immunocytometry Systems (BDIS), San Jose, CA, USA (Gratema et al, 1997), immediately after collection and at the time of reinfusion.

### RESULTS

A total of 40 patients, median age 55 years (range 19–70 years) were treated. Of these, 15 had gastric adenocarcinoma, nine had poorly differentiated cancer of unknown primary site, five had malignant melanoma, and nine had miscellaneous metastatic solid tumours. Pretreatment characteristics including median age, sex, WHO performance status, peripheral blood cell counts at entry and distribution of tumour types were essentially identical for the two patient cohorts (data not shown).

In cohort 1, 15 patients received 67 cycles at ifosfamide doses of 5.0, 5.5 and 6.0 g m$^{-2}$. Haematological toxicity is listed in Table 1. Dose-limiting toxicity (DLT) was reached at 6.0 g m$^{-2}$, with two out of six patients developing neutropenic fever.

Subsequently, in cohort 2, 25 patients received 87 cycles with ifosfamide at 5.5, 6.0, 6.5 and 7.0 g m$^{-2}$. Haematological toxicity is given in Table 2. Although no neutropenic fever was encountered, neither the frequency nor the duration (median 3 days in both cohorts) of grade 4 neutropenia and thrombocytopenia was reduced in comparison with cohort 1. Non-haematological toxicities and reasons for withdrawal are given in Table 3. Cumulative asthenia resulted in DLT in cohort 2 at 7.0 g m$^{-2}$.

As non-haematological toxicity was dose limiting in cohort 2, in order to make a better comparison between myelotoxicity encountered in cohort 1 and cohort 2, three additional patients at the ifosfamide dose of 6.0 g m$^{-2}$ were entered in both cohorts. All cycles of treatment per patient were taken into account for defining DLT, but for each patient who did not complete a minimum of three cycles, an additional patient was entered. This resulted in a total of nine patients and 35 cycles at dose level 6.0 g m$^{-2}$ in cohort 1, and 11 patients and 32 cycles at dose level 6.0 g m$^{-2}$. The data for these patients have been included in Tables 1–3.

Figure 1 shows the median number of CD34$^+$ cells in 500 ml of whole blood harvested at the time of cycles two to six. The median number of CD34$^+$ cells in 500 ml of whole blood after the first cycle (i.e. at start of cycle 2) was 1.15 × 10$^6$ kg$^{-1}$ body weight. This number was significantly greater after the second cycle (2.06 × 10$^6$ kg$^{-1}$, $P$=0.01) and then gradually decreased after cycles three to six. Although there was a wide range in the percentual difference

**Figure 1** Numbers of CD34 cells × 10$^6$ kg$^{-1}$ body weight in 500 ml of whole blood harvested at cycles two to six in numbers of CD34$^+$ cells after 51–55 h storage at 4°C of – 118% to +41% (2.5–97.5 percentile), there was no median decrease (in fact, there was a small increase) in the number of CD34$^+$ cells of +10.9%. (The small median increase during storage might be explained by further maturation of progenitor cells with initially low levels of CD34$^+$ expression).

### DISCUSSION

There is a rationale for delivering dose-intensified chemotherapy during repetitive cycles (Gurney et al, 1993). With the technical ability to increase the numbers of PBPCs in peripheral blood with G-CSF and chemotherapy, the approach of multiple cycles of dose-intensive chemotherapy each to be followed by progenitor cell reinfusion has become possible. For this purpose two different methods are being investigated. One technique is the harvest of progenitor cells by large-volume apheresis after conventionally dosed chemotherapy and cryopreserving the harvest in separate amounts, enabling progenitor cell support after each of subsequent dose-intensive cycles of chemotherapy (Benjamin et al, 1995; Leyvraz et al, 1995; Vahdat et al, 1995; Rodenhuis et al, 1996).

The second approach is the use of progenitor cells collected before each cycle of chemotherapy, which are kept refrigerated until the chemotherapy has been administered and cells can be reinfused. In addition, there is evidence that sufficient numbers of progenitor cells can be harvested in relatively small amounts of whole blood (Ossenkoppele et al, 1994, 1996; Pettengell et al, 1994, 1995). This approach would appear simple and cost-effective as it does neither require apheresis equipment nor cryopreservation of the cells. Furthermore, there is evidence of similar or even greater viability and colony-forming potential of short-term refrigerated cells compared with cryopreserved cells (Preti et al, 1994).

We studied the latter approach using progenitor cells present in a collection of G-CSF and 500 ml of chemotherapy-'primed' autologous whole blood in a 2-weekly dose-intensified cisplatin–ifosfamide regimen. Although we were able to harvest significant numbers of $CD34^+$ cells, especially during the initial cycles of chemotherapy, the reinfusion of these cells did not translate into a measurable reduction of the myelotoxic effects by the chemotherapy. It could be argued, however, that despite the increased dose of ifosfamide by 30% in cohort 2, the myelotoxicity with whole blood PBPCs at these dose levels of 6.5 and 7.0 g $m^{-2}$ was very similar to that at the lower dose levels without PBPCs. Also no neutropenic fever was encountered in cohort 2.

Nonetheless, the reconstitutive potential of PBPCs in whole blood in this schedule appears limited. The explanation for this cannot be found in technical aspects, as the harvesting technique, the medium and the duration of storage at 4°C were the same as applied by two investigators who reported on the use of PBPCs in whole blood (Ossenkoppele et al, 1994, 1996; Pettengell et al, 1995). The same holds true for the number of progenitor cells collected and the percentage of viable cells remaining after storage. Although the numbers of $CD34^+$ cells collected in 500 ml of blood in this study were slightly less than reported in the other studies in 500–750 ml of whole blood, the median number of $CD34^+$ cells of $1.15 \times 10^6$ $kg^{-1}$ at the second cycle and $2.06 \times 10^6$ $kg^{-1}$ at the third cycle should enable better identifiable effects on the haemopoietic reconstitution during the course of treatment. Also, the time interval of 20–24 h between the completion of ifosfamide infusion and reinfusion of PBPCs appears to be a sufficient wash-out period.

We have recently reported that the administration of G-CSF within 48 h before the start of chemotherapy has a detrimental effect on the bone marrow toxicity (de Wit et al, 1996). As, for successful PBPC collection, G-CSF needs to be continued until the time of collection, so as to keep the stem cells in the circulation, this finding is a clear disadvantage of the method of administering chemotherapy immediately after the harvest of PBPCs. In the present study, the last dose of G-CSF was administered approximately 24 h before the start of chemotherapy, thereby possibly resulting in harmful effects on the bone marrow. However, the continued administration of G-CSF in our study cannot be an explanation for the different findings by Pettengell et al (1995) and Ossenkoppele et al (1994, 1996), as in their studies G-CSF was also continued until or even within 24 h before the administration of chemotherapy (personal communication).

The alternative method of harvesting PBPCs by apheresis after one or two cycles of conventionally dosed chemotherapy and cryopreserving the cells in separate quantities for subsequent support of several of dose-intensive chemotherapy cycles appears a more attractive approach. We and others have demonstrated in patients with breast cancer and germ cell cancer that sufficient numbers of PBPCs can be collected by single apheresis to support three or four dose-intensive chemotherapy cycles (Bokemeyer et al, 1996; Rodenhuis et al, 1996).

We conclude that the method of combined bone marrow support by G-CSF and PBPCs in autologous whole blood collected before each cycle of a 2-weekly cisplatin–ifosfamide regimen does not result in clinically measurable reduced bone marrow toxicity, compared with what can be expected of support by G-CSF alone.

### REFERENCES

Benjamin RJ, Linsley L, Axelrod JD, Churchill WH, Sieff C, Shulman LN, Elias A, Ayash L, Malachowski ME, Uhl L, Gaynes L, McCauley M, Thompson L, Mazanet R, Antman K, Schnipper L, Tepler I, Antin JH and Wheeler C (1995) The collection and evaluation of peripheral blood progenitor cells sufficient for repetitive cycles of high-dose chemotherapy support. *Transfusion* **35**: 837–844

Bokemeyer C, Harstrick A, Metzner B, Beyer J, Ruther U, Berdel W, Casper J, Kuhrer I, Illiger HJ, Kempf B, Foller A, Holstein K, Derigs HG and Schmoll HJ (1996) Sequential high-dose-VIP-chemotherapy plus peripheral stem cell (PBSC) support for advanced germ cell cancer. *Ann Oncol* **7** (suppl.5): 55

De Wit R, Verweij J and Bontenbal M (1996) Adverse effect on bone marrow protection of prechemotherapy granulocyte colony-stimulating factor support. *J Natl Cancer Inst* **88**: 1393–1398

Dreger P, Haverlach T, Eckstein V, Jacobs S, Suttorp M, Löffler H, Müller-Ruchholtz W and Schmitz N (1994) G-CSF-mobilized peripheral blood progenitor cells for allogenic transplantation: safety kinetics of mobilization, and composition of the graft. *Br J Haematol* **87**: 609–613

Gratama JW, Kraan J, Levering W, Van Bockstaele DR, Rijkers GT and Van Der Schoot CE (1997) Analysis of variation in results of CD34+ hematopoietic progenitor cell enumeration in a multicenter study. *Cytometry* **30**: 109–117

Gurney H, Dodwell D, Thatcher N and Tattersall MHN (1993) Escalating drug delivery in cancer chemotherapy: a review of concepts and practice. Part 2. *Ann Oncol* **4**: 103–115

Leyvraz S, Ketterer N, Perey L, Bauer J, Vuichard P, Grob JP, Schneider P, Von Fliedner V, Lejeune F and Bachmann F (1995) Intensification of chemotherapy for the treatment of solid tumours: feasibility of a 3-fold increase in dose intensity with peripheral blood progenitor cells and granulocyte colony-stimulating factor. *Br J Cancer* **72**: 178–182

Ossenkoppele GJ, Jonkhoff AR, Huijgens PC, Nauta JJP, Van Der Hem KG, Drager AM and Langenhuijsen MMAC (1994) *Bone Marrow Transplant* **13**: 37–41

Ossenkoppele GJ, Schuurhuis GJ, Jonkhoff AR, Drager AM, Westra G, Oberink JW, Legdeur MCJC, De Kreuk AM, Zweegman S and Huigens PC (1996) G-CSF (filgrastim)-stimulated whole blood kept unprocessed at 4°C does support a BEAM-like regimen in bad-risk lymphoma. *Bone Marrow Transplant* **18**: 427–431

Pettengell R, Wol PJ, O'Connor DA, Dexter TM and Testa NG (1994) Viability of haemopoietic progenitors from whole blood, bone marrow and leukapheresis product: effects of storage media, temperature and time. *Bone Marrow Transplant* **14**: 703–709

Pettengell R, Woll PJ, Thatcher N, Dexter TM and Testa NG (1995) Multicyclic, dose-intensive chemotherapy, supported by sequential reinfusion of hematopoietic progenitors in whole blood. *J Clin Oncol* **13**: 148–156

Planting AST, Wit De R and Burg Van Der Mel (1996) Phase II study of a closely spaced ifosfamide–cisplatin schedule with the addition of G-CSF in advanced non-small-cell lung cancer and malignant melanoma. *Ann Oncol* **7**: 1080–1082

Preti RA, Razis E, Ciavarella D, Fan Y, Kuhns RE, Cook P, Wong G, Wuest DL and Ahmed T (1994) Clinical and laboratory comparison study of refrigerated and cryopreserved bone marrow for transplantation. *Bone Marrow Transplant* **13**: 253–260

Rodenhuis S, Westermann A, Holtkamp MJ, Nooijen WJ, Baars JW, Van Der Wall E, Slaper-Cortenbach ICM and Schornagel JH (1996) Feasibility of multiple courses of high-dose cyclophosphamide, thiotepa, and carboplatin for breast cancer or germ cell cancer. *J Clin Oncol* **14**: 1473–1483

Vahdat L, Raptis G, Fennelly D, Hamilton N, Reich L, Tiersten A, Harrison M, Hudis C, Moore M, Yao TJ, Norton L and Crown J (1995) Rapidly cycled courses of high-dose alkylating agents supported by filgrastim and peripheral blood progenitor cells in patients with metastatic breast cancer. *Clin Cancer Res* **1**: 1267–1273

© Cancer Research Campaign 1998